# A quantitative analysis of hydraulic interaction processes in stream-aquifer systems

**DOI:** 10.1038/srep19876

**Published:** 2016-01-28

**Authors:** Wenke Wang, Zhenxue Dai, Yaqian Zhao, Junting Li, Lei Duan, Zhoufeng Wang, Lin Zhu

**Affiliations:** 1 Key Laboratory of Subsurface Hydrology and Ecological Effects in Arid Region, Ministry of Education, Chang’an University, Xian, 710054, P.R. China; 2 Earth and Environmental Sciences Division, Los Alamos National Laboratory, Los Alamos, NM 87545, USA; 3 UCD Dooge Centre for Water Resources Research, School of Civil, Structural and Environmental Engineering, University College Dublin, Belfield, Dublin 4, Ireland; 4 College of Resources Environment and Tourism, Capital Normal University, Beijing 100048, China

## Abstract

The hydraulic relationship between the stream and aquifer can be altered from hydraulic connection to disconnection when the pumping rate exceeds the maximum seepage flux of the streambed. This study proposes to quantitatively analyze the physical processes of stream-aquifer systems from connection to disconnection. A free water table equation is adopted to clarify under what conditions a stream starts to separate hydraulically from an aquifer. Both the theoretical analysis and laboratory tests have demonstrated that the hydraulic connectedness of the stream-aquifer system can reach a critical disconnection state when the horizontal hydraulic gradient at the free water surface is equal to zero and the vertical is equal to 1. A boundary-value problem for movement of the critical point of disconnection is established for an analytical solution of the inverted water table movement beneath the stream. The result indicates that the maximum distance or thickness of the inverted water table is equal to the water depth in the stream, and at a steady state of disconnection, the maximum hydraulic gradient at the streambed center is 2. This study helps us to understand the hydraulic phenomena of water flow near streams and accurately assess surface water and groundwater resources.

Many streams in arid and semiarid areas are hydraulically disconnected from underlying aquifers^1^. When groundwater is pumped from near-stream aquifers for water supply in these areas, hydraulic disconnections may occur between streams and aquifers. For several decades, researchers have been modeling the interactions between streams and aquifers by both analytical^2^,^3^,^4^,^5^,^6^ and numerical^7^,^8^,^9^,^10^,^11^ methods. More recently, complex studies on stream-aquifer interactions with heterogeneous stream beds and variably saturated models are found in published reports^12^,^13^,^14^,^15^,^16^.

Generally, once a stream disconnects from the underlying aquifer, an unsaturated zone develops between the stream and aquifer^16^,^17^,^18^. Brunner *et al*.^1^ discussed the influence of conceptual assumptions on simulation results of the interaction between streams and groundwater by using off-the-shelf software. More research work attempted to address the impact of the unsaturated zone on groundwater recharge^7^,^12^,^13^,^19^,^20^,^21^,^22^,^23^,^24^. Stephens^25^ conceptually defined an inverted water table of the hydraulic disconnection in his book, but he did not give any further mathematical description of the concept and features. Wang *et al*.^18^ experimentally explored the physical processes from hydraulic connection to disconnection where a partially penetrated stream-aquifer-well system was built in a sandbox in the laboratory. The experiment results indicate that an inverted water table always appears below the stream when the hydraulic disconnection occurs, whether the streambed consists of a clogging layer or not. In such cases, the disconnected stream-aquifer system mainly includes the stream, inverted saturated zone beneath the stream, unsaturated zone, and saturated groundwater zone. Therefore, both the inverted water table and the unsaturated flow are key elements in stream-aquifer systems. However, from literature, modeling and evaluation of the impact of the conceptual assumptions on the properties of the hydraulic disconnection often ignored the inverted water table below the streambed^17^, which has a large impact on stream seepage (or infiltration) fluxes to groundwater. Furthermore, Sophocleous^26^ stated that most models are incapable of dealing with the local-scale hydraulic processes at the interface boundaries between the stream and aquifer.

This paper presents a mathematical description of the hydraulic processes of the stream-aquifer system with a groundwater pumping well near the stream in a symmetrical flow system. Our special focus is on the following issues: 1) under what conditions does the stream start to disconnect from the aquifer? 2) what happens between the stream and aquifer after the stream disconnects from the aquifer? 3) what equations can be used to appropriately describe the movement of the critical point of disconnection below the streambed? 4) what hydraulic gradients at the center of the streambed can be reached at a steady state after disconnection? Thereafter, the application of the numerical method is presented for a free water-table equation and the analytic approach of the boundary-value problem of the inverted water table is discussed to explore the specific hydraulic processes. Finally, a validation of the mathematical analysis is conducted against an experimental result to summarize and conclude this study.

## Results

This study examined the physical processes of the hydraulic connectedness of the stream-aquifer systems from connection to disconnection due to groundwater pumping (or drainage) near the stream by using the analytic numerical method combined with the sandbox experiments. The main results from this study are as follows:

1. When a stream disconnects from aquifers, a stream-aquifer system consists of the stream, inverted water table beneath stream, unsaturated zone, and saturated groundwater zone. The water table drawdown due to intensive pumping (or drainage) near the stream can potentially reduce saturations in the aquifer between the well and stream. The relationship between the stream and aquifer would evolve from the hydraulic connection to disconnection if the pumping intensity exceeds the maximum seepage capacity of the stream under a given streambed condition.
2. When hydraulic heads at the free water surface satisfy 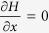 and 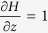 (where *H* is hydraulic heads, and 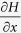 and 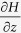 are hydraulic gradients in the horizontal (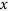) and vertical (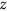) directions, respectively) for a symmetrical stream-aquifer system, the hydraulic connectedness between the stream and aquifer is at a critical disconnection state.
3. From the simplified boundary-value problem of the inverted water table movement, we deduce that the maximum distance (or vertical length) of the inverted water table beneath the stream is the same as the water depth in the stream. The maximum rate of stream infiltration (per unit time per area) is equal to two times of the hydraulic conductivity of the streambed. This result implies that the maximum hydraulic gradient at the streambed center is 2 when the disconnection occurs.
4. The results of the sandbox experiment, a recent field test and the finite analytic numerical solution are consistent with the mathematical analysis results of the hydraulic disconnection processes. These results will help us to understand the hydraulic phenomena of variably saturated flow near streams and to accurately assess surface water and groundwater resources.

## Discussion

### The evolution of hydraulic gradients in the center of the streambed from connection to disconnection

**Figure 1:**
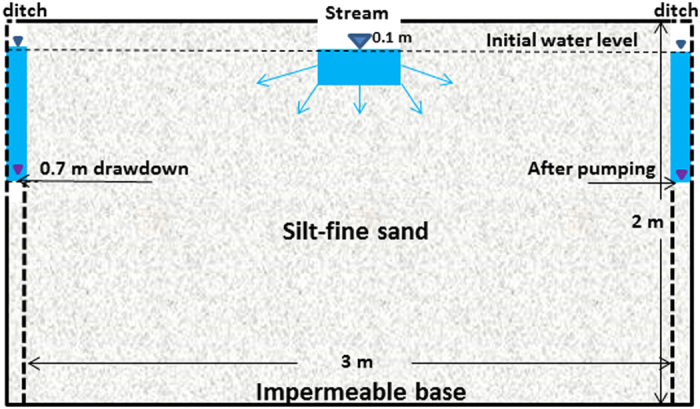
The conceptual model of a lab-scale stream-aquifer system for explaining the evolution of hydraulic gradients under the streambed.

To derive our mathematical analysis methods, we develop a lab-scale stream-aquifer system as an example for explaining the evolution of hydraulic gradients under the streambed (Fig. 1), where the hydraulic relationship of the stream-aquifer system evolves from connection to disconnection. The flow domain is 3 m in length, 2 m in height and 1 m in width. The stream is 0.2 m in width and is located along the center line of the flow domain. The water depth in the stream is 0.1 m. There are two ditches (which function equivalently as two pumping wells) located equidistant 1.4 m from the stream bank on each side. The stream stage is initially the same as the water level in both ditches. When the water levels in both ditches were suddenly declined to 0.7 m and then remained constant during the period of simulation, the stream-aquifer system were evaluated from connection to disconnection and finally reached a steady state. The porous medium in the aquifer is silt-fine sand and the corresponding parameters for the unsaturated water flow are summarized in Table 1. The sediments of streambed are the same as the medium in the aquifer.

A finite analytic numerical method developed by Dai *et al*.^27^, Wang *et al*.^28^, and Zhang *et al*.^29^ for the saturated-unsaturated flow simulation was adopted in the validation. Relative conductivity functions were broadly used by many investigators to simulate water flow in variably saturated zones^30^,^31^,^32^,^33^,^34^. For simplicity, here, we assume that the soil hydraulic conductivity follows the exponential relative conductivity model^35^.


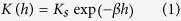


where, *h* is the soil-water pressure head (m); *K*(*h*) is the unsaturated hydraulic conductivity (md^−1^); 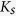 is the saturated hydraulic conductivity (md^−1^); 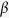 is an arbitrary constant. The value of 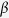 can be estimated through curve fitting of experimental data, and it is 6.6 in this study.

The ground surface boundary condition of two sides of the stream was assumed to be the boundary of the prescribed negative pressure head with four scenarios. The first scenario assigns a pressure of −2 m while the second and third scenarios are −1 m and −0.5 m, respectively. In the fourth scenario, the pressure of the ground surface for two sides of stream changes with a uncertain interval between 0 m at stream bank and −2 m at the ground surface edge of two sides of the stream.

The boundary condition of the stream is a prescribed pressure head, which is the same as the water depth in the stream. The boundary conditions of the saturated zones on both sides of the ditches are also a prescribed pressure head, which is equal to the actual water head in the ditches. Accordingly, the boundary condition of the unsaturated zones on both sides of the ditches from the ground to the water surface in the ditches is a prescribed negative pressure head, which is given by linear interpolation between negative pressure of the ground and zero pressure.

**Figure 2:**
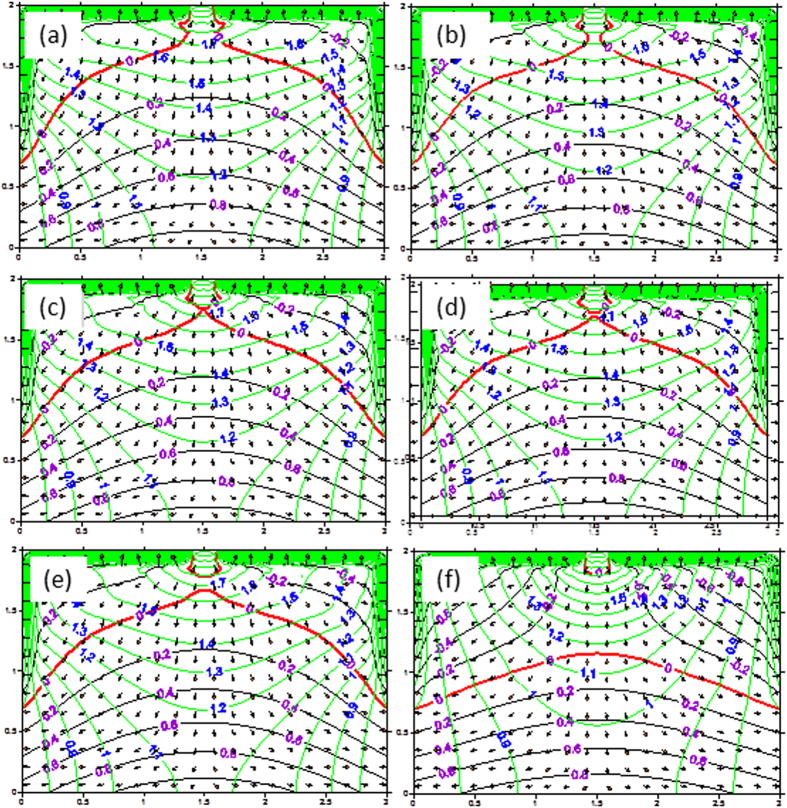
The simulated seepage fields for the ground surface boundary condition with 2 m negative pressure at time of : (**a**) 0.012d, (**b**) 0.0185d, (**c**) 0.0198d, (**d**) 0.02d, (**e**) 0.022d, and (**f**) 2d. (The pressure water heads are in black and red. The red line represents zero pressure which indicates the water table and the inverted water table curves respectively. Total water head is in green and flow directions are indicated in arrow).

Figure 2a illustrates the evolution of the pressure water head and total water head of seepage field with time for the ground surface boundary condition of 2 m negative pressure simulated by the finite analytic numerical method of the saturated-unsaturated flow. Figure 2b shows the simulated patterns of the pressure water head and total water head of seepage field at steady-state seepage in scenarios 1, 2, 3, and 4 of the ground surface boundary conditions.

**Figure 3:**
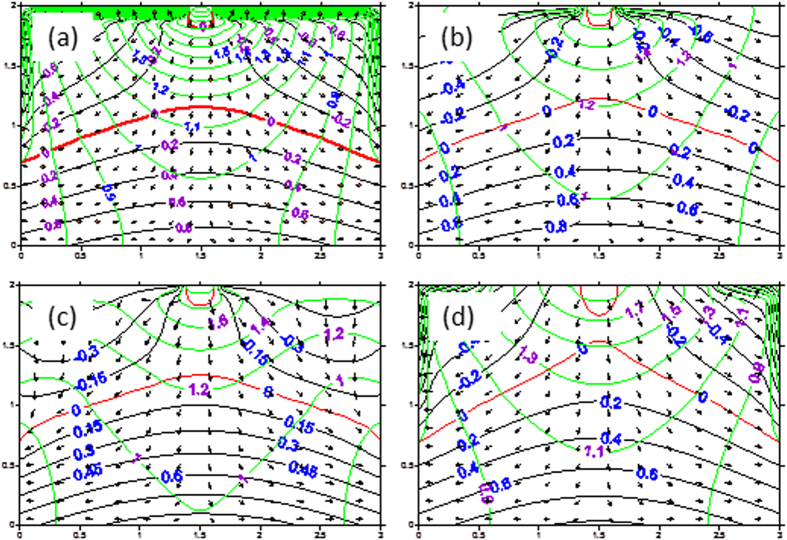
The behaviors of steady state seepage fields for different ground surface boundary conditions after disconnection ((**a**–**d**) correspond to scenarios of 1, 2, 3, and 4, respectively).

The numerical simulation results computed from the saturated-unsaturated flow model^27^,^28^,^29^ clearly show that, as long as the rate of drainage in the ditches exceeds the seepage capacity of the stream under a given streambed condition, the relationship between the stream and aquifer can be evolved from the hydraulic connection to disconnection (Fig. 3). The results of the numerical simulations are consistent with those of the laboratory experiments^18^. This demonstrates that the numerical approach proposed in this study can be used to describe the stream-aquifer relationship, which includes three hydrologic steps from connection to disconnection: connection (Fig. 2a,b), critical disconnection (Fig. 2c), and entire disconnection (Fig. 2e,f). Note that Lamontagne *et al*.^15^ developed a nomogram to estimate the height of the groundwater mound without the limiting assumption of horizontal flow. With their approach, the steep gradients right in the center of the mound can be simulated and their results are the same as those shown in Fig. 2c,d,f in this study. By inspecting the flow patterns of the stream-aquifer system for the four ground surface boundary conditions in Fig. 3a,d, we can see that the boundary conditions have a great impact not only on the flow patterns or hydraulic gradients of the stream-aquifer system, but also on the river recharge to aquifer. The hydraulic gradients at the streambed can be used to assess its influence on the stream recharge to the underlying aquifer.

**Figure 4:**
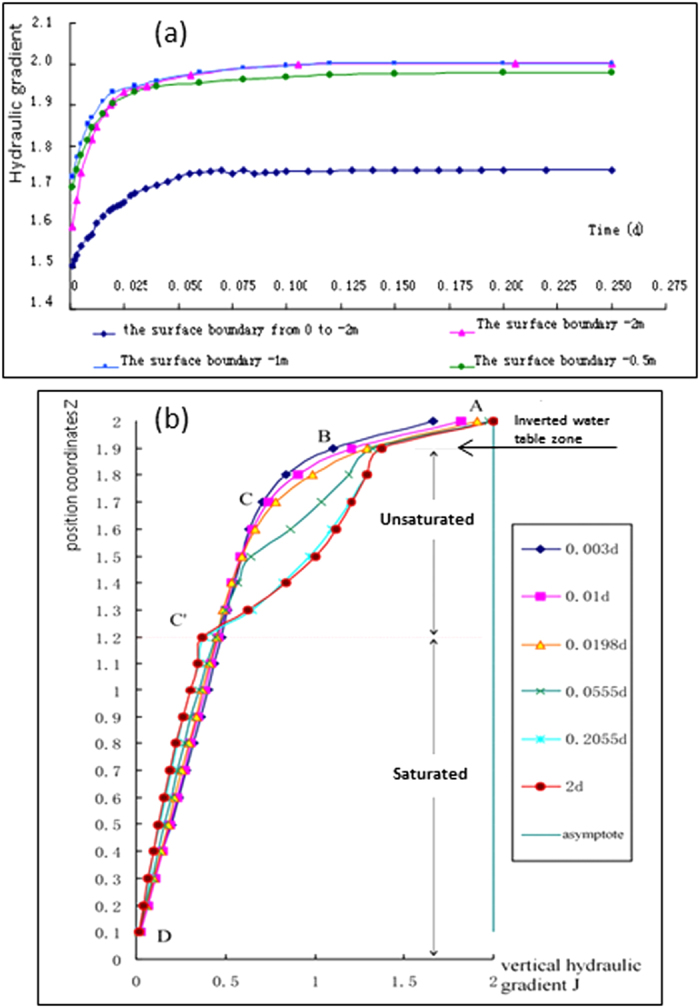
The hydraulic gradient variations with time at the center of the streambed for different ground boundary conditions (**a**) and the hydraulic gradient variations with time (from hydraulic connection to disconnection) on the symmetrical line (**b**).

Figure 4a describes the variations of the hydraulic gradients at the center of the streambed with stream water depth of 0.1 m, ditch discharge level of 0.7 m, and four different ground surface boundary conditions. By assuming a ground surface pressure of −2 m, we analyze the change in the hydraulic gradients from the hydraulic connection to disconnection. Figure 4a shows that the hydraulic gradient quickly increases during the period of 0 to 0.0198 d. During that time the stream keeps connected with the aquifer. When time reaches 0.0198 d, the relationship between stream and aquifer becomes the critical disconnection state. At that time the curve of the hydraulic gradient shows an obvious turning point. From 0.0198 to 0.05 d the stream disconnects from the aquifer, and the hydraulic gradient increases slightly. From 0.05 to 0.125 d the hydraulic gradient shows very little change. After 0.125 d the flow of the stream-aquifer system reaches to a steady state, and the hydraulic gradient reaches to the maximum value (approximately 2 at the center of the streambed). Similar trend in the hydraulic gradient variation for the ground surface pressure of −1 m can be seen in Fig. 4a. When the ground surface pressure is assumed to be −0.5 m, the hydraulic gradient at the center of streambed is slightly less than 2 after disconnection. The reason is that there is some unsaturated water flowing into the stream-aquifer system from the ground surface boundary (see Fig. 3c). This reduces the river recharge to aquifer compared with the cases which have higher negative pressures. Therefore, the less of the negative pressure on the ground surface boundary, the more water flows into the aquifer from the ground surface boundary and much less hydraulic gradient at the center of streambed after disconnection (see Figs 3c,d and 4a). More significantly, the maximum capacity of stream recharge to aquifer per unit area per time is no more than 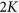
(*K* is the hydraulic conductivity of the streambed) when the stream disconnects from aquifer at the steady state. This result is consistent with that obtained by the analytic method.

### The hydraulic gradient variation with time on the symmetrical line

Figure 4b shows the time-variation of the hydraulic gradient on the symmetrical line from streambed center to impermeable base of aquifer from hydraulic connection to disconnection. The figure presents cases of a 0.1 m water depth in the stream, a 0.7 m discharge level in both of ditches, and a −2 m pressure at the ground surface, respectively. The curves with time 0.003 d and 0.01 d indicate the variation tendency of the hydraulic gradient on the symmetrical line while the curve with 0.0198 d shows the hydraulic gradient at the critical disconnection state. Accordingly, the curves with time 0.0555, 0.2055 and 2 days are the variation trend of the hydraulic gradient for disconnection state, especially the curve with time 2 days indicates an entire disconnection situation at the steady state.

As can be seen from Fig. 4b, no matter the stream is disconnected with aquifer or not, the time-variation of the hydraulic gradient on the symmetrical line from streambed center to impermeable base can be divided into three zones. The first zone (termed as the inverted water table zone) is located at the certain depth below streambed (see Fig. 4b section AB). The essential features of the first zone are: 1) The depth of the zone is nearly equal to stream water depth; 2) The time-variation of the hydraulic gradient shows a slightly growing trend from connection to disconnection; 3) The vertical hydraulic gradient is linear with the vertical coordinate distance *z*; 4) The vertical hydraulic gradient at the streambed center reaches 2 at the steady state after disconnection.

The second zone (termed as the saturated zone) is located at the certain height above the impermeable base (see Fig. 4b section C′D). The time-variation of vertical hydraulic gradient shows a slightly decreasing trend from connection to disconnection. The vertical hydraulic gradient is also linear with the vertical coordinate distance *z*, but with moderated slopes compared with that of the first zone.

The third zone (termed as the unsaturated zone) is located between the first and the second zones (see Fig. 4b section BC and BC′). There are several essential features in the seepage flow: 1) The vertical gradient has bigger amplitude of variation from connection to disconnection, especially under disconnection condition; 2) When stream disconnects from aquifer, there exists an unsaturated zone between the inverted zone and the saturation zone. Unsaturated flow in this zone dominates in the flow and transport processes of the stream-aquifer system. The nonlinear flow in the unsaturated area depends on the relative conductivity functions which are variable with moisture contents and hydraulic pressures. This may be the reason to cause a larger variation amplitude of the vertical hydraulic gradient in the unsaturated zone than that in the saturated zone; 3) The vertical hydraulic gradient in this zone is nonlinear with the vertical coordinate distance *z*.

### The variation of vertical hydraulic gradient at the free water surface of the regional water table

**Figure 5:**
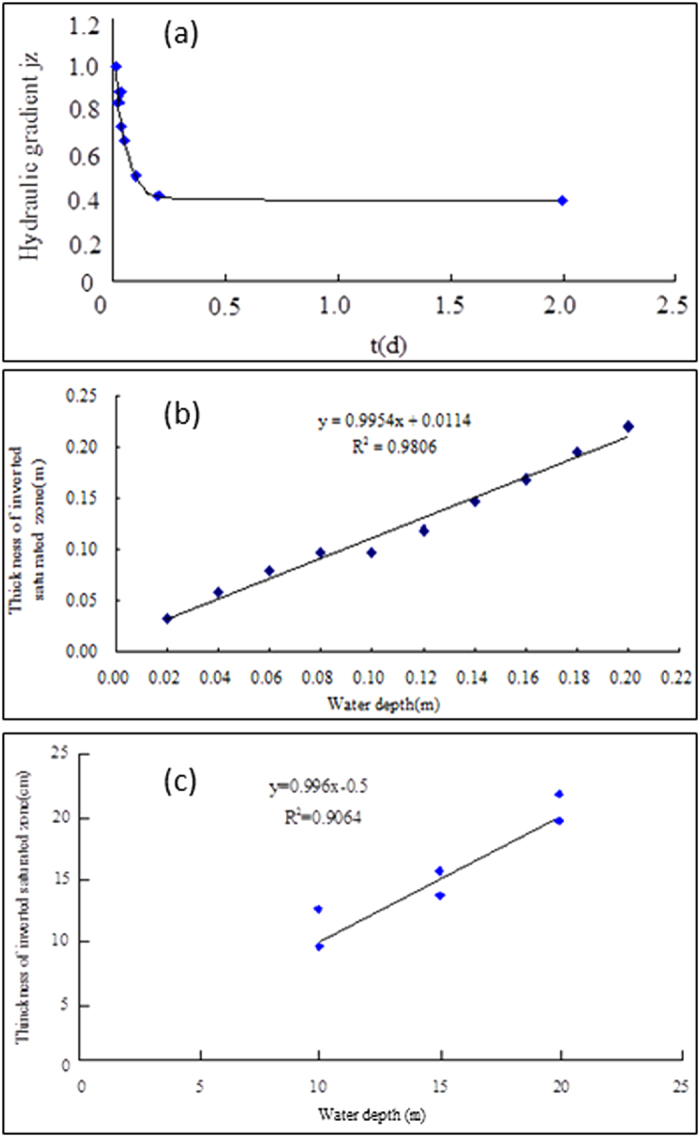
The time-variation of the vertical hydraulic gradient 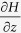 at the free water surface of regional water table on the symmetrical line from the critical disconnection to entire disconnection (**a**), the relationship between the maximum thickness of inverted water table zone below streambed and the water depth in stream at steady state for entire disconnection observed by the laboratory sandbox experiment (**b**), and by the finite analytic numerical method (**c**).

Figure 5a shows the time-variation of the vertical hydraulic gradient 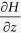 at the free water surface on the symmetrical line from the critical disconnection to the entire disconnection. The results were obtained by using the finite analytic numerical method under the conditions of 0.1 m water depth in the stream, 0.7 m discharge level in both of ditches, and −2 m pressure of the ground surface. The gradient 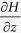 at the free water surface on the symmetrical line is equal to 1 for the critical disconnection at 0.0198 days. After this moment the gradient 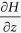 is gradually reduced because the ground-water mound of stream recharge drops in response to drainage from both sides of ditches. Interestingly, as discussed previously, when the hydraulic head at the free water surface meets 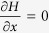 and 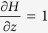 for a symmetrical stream-aquifer system, the hydraulic connectedness of the stream-aquifer reaches the critical disconnection state.

### The maximum thickness of the inverted water table zone beneath the stream

Figure 5b,c show that the maximum thickness of the inverted water table zone beneath the stream varies with the water depth in the stream at the steady state for entire disconnection by using the laboratory sandbox experiment and finite analytic numerical method, respectively. It can be seen that the higher the stream stage, the thicker the inverted water table zone. In addition, the slopes of the curves in Fig. 5b,c are nearly equal to 1. This implies that the maximum thickness of the inverted water table zone is the same as the water depth in the stream at the steady state for entire disconnection, which agrees with the above conclusion obtained by the analytic method.

**Figure 6:**
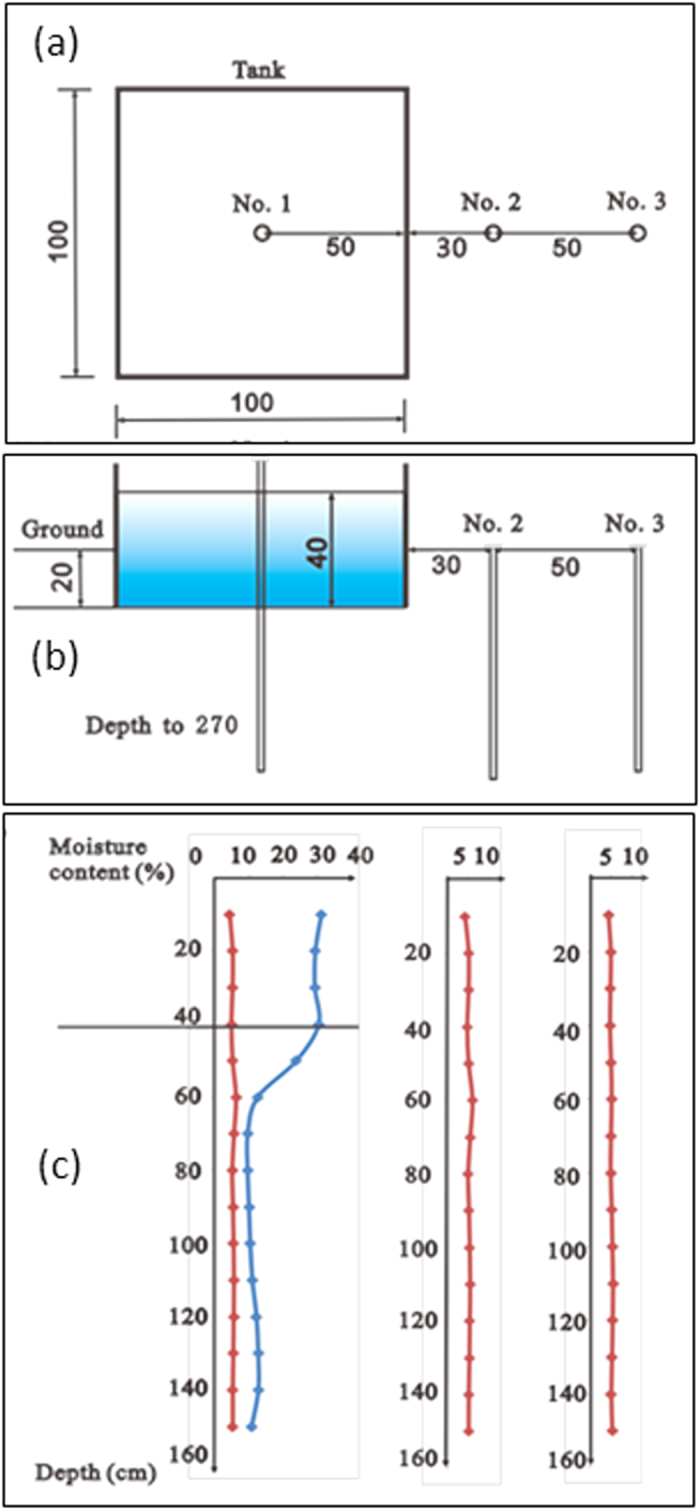
A sketch and the results of the field test. (Part (**a**) is the plane section while (**b**) is the cross section of the field test site, and (**c**) is the distributions of moisture contents in the three boreholes at different depths during the test).

In addition, a recent field test in the Ordos Basin, China, was designed to see if there is an inverted water table beneath the stream, and if its maximum thickness is equal to the water depth in the stream (at the steady state under the condition of streambed sediments being the same as the underlying aquifer materials). A site was selected in an area with uniform fine sand in the unsaturated zone, where the depth of groundwater is about 8 m according to observation wells near the testing site. A quadrate test tank (1 × 1 × 1 m^3^) held a bed of uniform fine sand. Three boreholes with a diameter of 40 mm located at the center line of the tank, 0.3 m and 0.5 m from another side of the tank, respectively, were drilled from the ground surface to 2.7 m deep. These boreholes were used to measure moisture contents by TDR during the test period. Several overflow holes are designed on one side of the tank, which are used to keep the artificial stream stage at a constant level with the tank. The height from the tank bottom (similar to streambed surface) to the overflow hole represents the simulated water depth in the stream. The moisture contents were firstly measured as the initial condition. Then water is added to the tank at a sufficient large rate so that the water in the tank is kept at a constant level (0.4 m in this test). The saturated water content of fine sand is 0.322 cm^3^/cm^3^. The moisture contents were measured from three holes at 10–20 minutes intervals over the entire test period. The test was stopped until the steady state was reached. A sketch of the field test is shown in Fig. 6(a,b). Figure 6c shows the distributions of the moisture contents in the three boreholes at different depths. It can be seen that there exists a saturated zone below the tank bottom in the centre borehole at the steady state. The thickness of the saturated zone below the tank bottom in the centre borehole is almost equal to the water depth in the tank, which validates the result of Eq. (19) in the method section. This is equivalent to the maximum hydraulic gradient with a value of 2 at the streambed under steady-state condition for the disconnected stream. The measured moisture contents in another two boreholes remain no change during the test.

## Methods

### The prerequisite of the total water head at the water table for the critical disconnection condition

**Figure 7:**
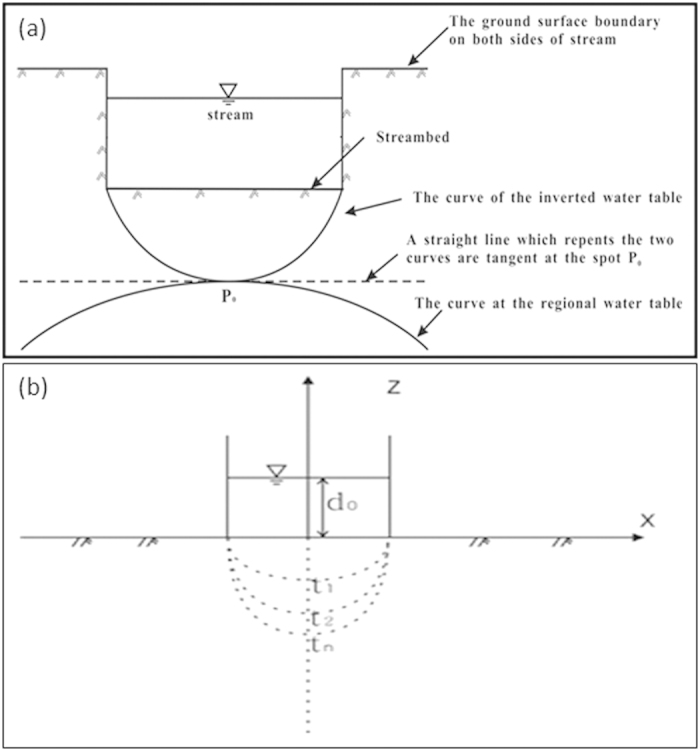
The water-table curves in the vertical profile at the critical disconnection state. (**a**) and the inverted water table below streambed at different times for a symmetrical stream-aquifer system (**b**).

Brunner *et al*.^12^,^13^ and Wang *et al*.^18^ have discussed the hydrogeological controls of disconnection and transient effect during the transition from connection to disconnection in great detail. The point when the water-table curve starts to divide into two parts can be termed as the critical disconnection point. At this point, the water-table curve converges at a spot 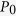 (see Fig. 7a) on the central axis below the streambed for a symmetrical flow system. There are two water-table curves at the critical disconnection point (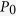) in the vertical profile. One is located above 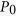 spot which is called the inverted water table^18^, and the other is called the regional water table which is located below 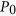 spot. Actually, the differences of the different water table curves are mainly the variations of the hydraulic gradients along the water table curves, which cause changes in the geometric shape of water table curves.

From the theory of the differential geometry, 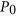 spot at the critical disconnection state is a singular point on the vertical profile of the water table curve. As long as the first and second-order derivatives of the curve exist, the prerequisite can be obtained while the horizontal and vertical hydraulic gradients of the water table curve in the vertical profile reaches certain values at the critical disconnection state.

The free water surface equation can be used to estimate the prerequisite when 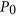 is reached. The equation for an isotropic, homogeneous, unconfined aquifer in the vertical profile at the steady state can be expressed as^36^


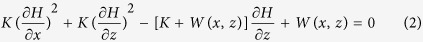


where, 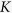 is the hydraulic conductivity (m/d), 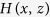 is the total water head in the vertical profile (m), and 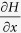 and 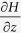 are the hydraulic gradients in the horizontal (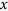) and vertical (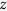) directions at the water table curve, respectively, and 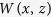 is a vertical exchange rate per unit area per unit time at the water table for a given discharge level at the steady state (m^3^/m^2^.d). Eq. (2) can be reorganized as


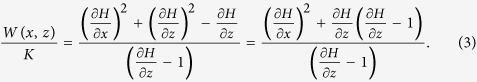


The left hand in Eq. (3) represents the ratio of the vertical exchange at the water table to the hydraulic conductivity of the aquifer for a given steady-state discharge level in the ditch. The right hand, which is only relevant to 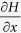 and 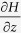, represents variations of hydraulic gradients in the 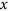 and 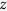 directions at the water table. Both sides of Eq. (3) are dimensionless. It is known from the calculus, once 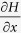 and 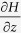 are determined, the position of water table and the shape of water table curve in the vertical profile can be known in conjunction with the boundary condition (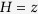). So the right hand of Eq. (3) actually describes variations of the water table curves in the vertical profile, which is driven by the term of 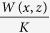. As 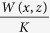 varies with the discharge level in the ditch, the shape of water table curve changes accordingly. In addition, Eq. (3) can also be used to determine a vertical water exchange, i.e. 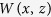 of the free water surface at steady state in the ditch. Actually, 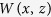 is the water balance at the water table and


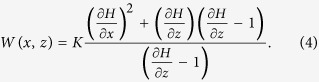


Eq. (4) can be used to analyze variations of the water table curves at different discharge levels in the ditch. Therefore, a new function of 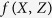 is defined, which is the shape function of the water table curve in the 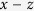 profile. It describes the shape of the water table curve at steady state for given boundary conditions in a flow domain. 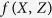 can be written as


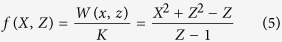


where, 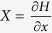 and 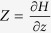. It is expected to use the shape function to sort out the important query: under what conditions the stream starts to disconnect from aquifer. For doing so, the partial derivatives and the mixed partial derivatives respect to variables of 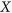 and 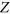 of the function 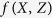 are conducted as:


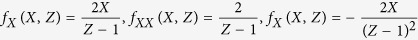



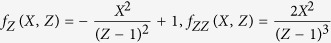


Letting 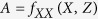, 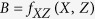 and 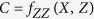, we have


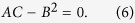


According to differentiable function properties and criterion^37^,^38^, Equation (6) indicates that the point of *X* *=* *0* and *Z* *=* *1* is a singularity point marked as 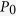 on the water table curve defined by 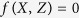, where the water table curve starts to separate into two curves (one is the inverted water table and the other is the regional groundwater table), and the point 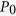 is the critical point for disconnection. Therefore, 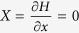 and 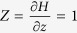 are the prerequisite conditions for hydraulic disconnection, which corresponds to a general solution of 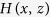 = *C*_*1*_ and 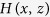 = *z* + *C*_*2*_, where *C*_*1*_ and *C*_*2*_ are constants. If all of the initial-boundary conditions are known, we may compute the values of the two constants.

As such, the two free water table curves exactly converge at a spot 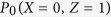 on the central axis below the streambed for a symmetrical stream-aquifer system. When the inverted water table and the regional groundwater table are tangent at 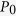 point, the exchange flux between the two free water tables is equal to 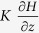. This is consistent with the Darcy’s law. Above mathematical derivations demonstrate that the hydraulic connectedness of the stream-aquifer system is at the critical disconnection point when the hydraulic head at the water table curve satisfies 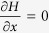 and 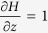 for a symmetrical stream-aquifer system, in which the aquifer is an isotropic and homogeneous system with two ditches (or pumping wells) located equidistantly (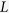) on each side of the stream with an equal drainage (or pumping) rate.

### The boundary-value problem for the inverted water table and its movement in a hydraulically disconnected stream-aquifer system

There exists an inverted water table below the streambed when the stream disconnects hydraulically from the underlying aquifer^12^,^13^,^18^. In order to derive the equations of the inverted water table movement and its properties, several assumptions can be made for a reverse disconnection case: (1) The stream is initially disconnected from an aquifer, where the groundwater depth is much deeper than the streambed. The sediments of streambed are the same as aquifer materials and are isotropic and homogeneous. There is no clogging layer in the streambed. (2) The stream channel is dry at time *t* *=* *0*, and then the water flow occurs through channel with depth 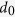. For a period of time, an inverted water table is gradually formed below the streambed (Fig. 7b). This situation usually exists in the arid areas where ephemeral streams flow through alluvial fans and the depth of groundwater can be more than 100 m in the upstream areas and 3–5 m in the downstream areas for the stream disconnected from groundwater^39^. To obtain the boundary-value problem of the inverted water table (Here we take the streambed center as the original point of the horizontal and vertical coordinates), the free water surface equation of the inverted water table should be determined firstly at the unsteady state, which is one of the boundary conditions in the boundary-value problem.

Take *z* as the vertical coordinate distance and the upward represents positive, and let 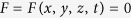 represent the implicit equation at the free water surface of the inverted water table. The total water head on the free water surface is equal to the vertical coordinate of any point on the free surface of the inverted water table, i.e. 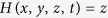


Similarly, for a homogeneous and isotropic medium, the free water surface equation of the inverted water table can be written as^36^,


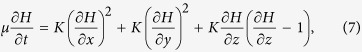


Here, we do not intend to solve the Equation (7), but try to use it to estimate the movement velocity of the inverted water table and to explore the relationship of the related parameters at the a disconnection state. When x, y, and z are close to the critical point of disconnection, or a singularity point at the inverted water table, we may assume that the second order of derivatives 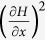, 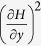 and 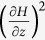
can be neglected in Eq. (7) because there terms are approached to zero when x, y, and z are close to the critical point. Thus, Eq. (7) can be approximately expressed as:


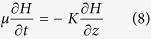


Here, 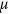 is specific yield. Because of symmetry, only the symmetrical line from the streambed center to aquifer bottom in Fig. 7b needs to be considered. This simplifies the problem (7) to be a one-dimensional equation to describe the movement of the critical point of disconnection. If the water depth in the stream is a constant 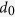 for a period of time, the flow system near the critical point can be described mathematically as follows.


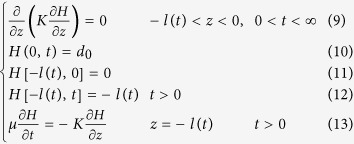


The term 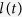 represents the moving distance of the inverted water table front at time *t* in the boundary-value problem of Eq. (9)–(13).

Integrating Eq. (9) and using the boundary condition (11), (12) and (13), and noting that 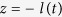 and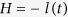, we obtain Eq. (14) as


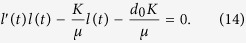


The nonlinear differential equation (14) represents a mathematical relationship of the moving velocity and distance for the inverted water table front. The analytic solution of the differential Equation (14) subjecting to Eqs. (10) to (13) is


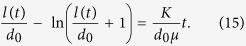


Letting 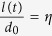, the front movement equation for the critical point in the symmetrical line can be expressed by


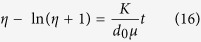


Eq. (15) and (16) indicate that (1) Both of 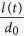 and 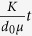 are dimensionless factors. Both sides in Eq. (16) represent dimensional homogeneity; (2) Because upward in z direction is positive, it appears 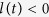, or the inverted water table moves downward to the regional groundwater table. If 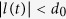, both sides in Eq. (16) have the same mark symbol sign; (3) If 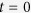, 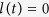, 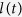 represents the initial condition in Eq. (11); and (5) In the steady-state condition, the velocity of the free water surface movement is 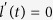, and by using Eqs (14) and (16) becomes:


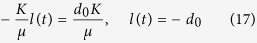


Therefore, under the one-dimension and steady-state circumstance, the front moving maximum depth of the inverted water table zone is equal to the stream water depth (here, it does not contain the capillary height rise 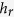, if it contains 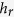, then 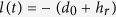), and below this distance there is a unsaturated zone until the regional groundwater table.

### The maximum hydraulic gradient at the streambed under a steady-state condition

By combining Darcy’s Law and the free water surface movement Eq. (17), the downward flow velocity through streambed into the inverted water table zone at steady-state condition is:


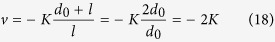


Eq. (18) clearly indicates that when a stream is disconnected with groundwater level under a steady-state condition, the maximum critical velocity for the stream recharging the unsaturated zone (per unit area per unit time) is not larger than two times of the streambed hydraulic conductivity. This result implies that the maximum hydraulic gradient in the center of the streambed is 2 for the disconnected stream under a steady-state condition.

## Additional Information

**How to cite this article**: Wang, W. *et al*. A quantitative analysis of hydraulic interaction processes in stream-aquifer systems. *Sci. Rep.* **6**, 19876; doi: 10.1038/srep19876 (2016).

## Figures and Tables

**Table 1:** The parameters of the silt-fine sand for unsaturated and saturated conditions.

**Medium type**	**Residual water content (*****θr***)	**Saturated water content (*****θs***)	**Saturated hydraulic conductivity k**_**s**_**(cm/h)**
Silt- fine sand	0.045	0.43	7.13
